# Blood-Letting Not Necessary in Pneumonia

**Published:** 1866-02

**Authors:** D. W. Young

**Affiliations:** Aurora, Ill.


ARTICLE IV.

### BLOOD-LETTING NOT NECESSARY IN PNEUMONIA.

By D. W. YOUNG, M.D., of Aurora, Ill.

An address read before the Aurora City Medical Association, at its annual meeting, Friday evening, January 12th, 1866, by the President of the Association.

Gentlemen of the Association:—Since you, at our last meeting, had the hardihood to impose upon me the task of writing something for the consideration of the Association this evening, I have determined to, in turn, impose upon your time by offering a few, perhaps illy-digested, reasons why I deem blood-letting not necessary in pneumonia. I shall not do so, however, expecting to answer fully the able paper and arguments presented at our last meeting, by my friend, Dr. S. R. Millard, favoring this remedy in this disease. Nevertheless, I deem it my duty to try and give reasons for the belief that is in me.

First. What is pneumonia? Is it a disease of function or structure, or both? All diseases consist in a change from the natural condition of the function or structure of the body. It is quite obvious, therefore, that we cannot obtain an accurate knowledge of the nature of disease until we have carefully studied the component parts of which it is made. As the anatomist or physiologist examines structures and functions by separating or analyzing them into their constituent parts before he enters on their contemplation as a whole, so must the pathologist carefully study and analyze the constituent parts or elements of disease, before he attempts to understand their combinations, consequences, or applies his remedies.

In pneumonia, we first have an intense injection of the capillaries of the lung. The blood is dammed up and the heart's action increased in consequence. The substances of the lung, the cells, and parenchyma are gorged with blood or bloody serum. The functions of respiration and circulation are impeded, accelerated, and otherwise changed from their normal standard. There is too much blood and not enough air in the lung, *without any actual disorganization.* The temperature of the body is exalted. The patient takes many forced inspirations, sighs, or gasps, and the breathing is quickened on the least exertion. The functions of the nervous system are also impaired, by reason of the defective oxygenation of the blood; the skin soon assumes a blue, unhealthy look, denoting that the aëration of the blood is imperfectly performed. The respiration is hurried and labored, with pain at each inspiration. The skin is dry and hot; pulse full and quick; bowels inclined to constipation; urine scanty and high-colored; some cough; slight expectoration, which is streaked with blood. There is intense congestion of the lung, which constitutes the first stage of the disease—the period of high febrile action.

If this engorged and congested condition be allowed to continue, the lung, after a time, undergoes further alterations. It still remains congested and red, externally and within; but it crepitates no longer under pressure; it now contains no air. The spongy character of the organ is lost; there is an interstitial deposit of lymph in the parietes of portions of the bronchial tubes and vesicles. The second stage of the disease, hepatization, is inaugurated, which, if permitted to remain and proceed, soon produces still further changes. The air-cells are obliterated; the aëration of the blood is still more improperly and imperfectly performed; the pulmonary circulation is, to a certain extent, arrested. The blood is changed; it no longer passes freely and properly over the air-cells of the lung, to be exposed to the aërating action of the atmosphere. The hemato-globuline is changed, by reason of the defective oxygenation, and it is sent back through the systemic circulation, loaded with carbonic acid, and deficient in oxygen, and, as a consequence, the brain, nervous and muscular systems robbed of their accustomed and essential stimulus; the temperature of the body is lowered; the vital powers of the system reduced; the products of the inflammation changed from plastic to aplastic, and disorganization commences; the interstitial lymph gives place to a purulent fluid; and the third stage of the disease, gray hepatization or softening, is at hand. In relation to the causes of pneumonia, I shall offer nothing at this time, my only object in this paper, being to discuss the propriety of blood-letting as its remedy.

What are the indications for treatment in pneumonia? What the pathological condition to be remedied? In the first stage of the disease, we have an injection of the capillaries of the lung, soon followed by engorgement and infiltration of the cells and parenchyma. There is too much blood in the organ; as a consequence, respiration and pulmonary circulation are impeded. The question arises, What is the cause of there being too much blood in the lung? Is it because there is too much blood in the system? I apprehend not, as a general rule. On the contrary, we quite frequently meet with the most violent attacks in anæmic subjects—subjects who are deficient both in quantity and quality of blood. In these cases, at least, we must look for some other cause than the quantity of blood on hand. The object to be accomplished in this stage, is to unload the lung and its capillaries of the surplus blood; to equalize the circulation, and lessen the heart's action, in order that the blood shall not again be pumped back upon the lung with too much force.

Now, how are these very desirable objects to be attained? Dr. Millard says, by bleeding. I object. I contend that is too expensive to the constitution, *and unnecessary.* I hold that we are possessed of other means. ess expensive and equally efficacious. Why is blood-letting expensive? What remote effect does the loss of blood produce upon the constitution? Does it lessen the quantity in circulation? Yes, but only for a short time. Does it change the quality of the blood? Yes, very materially. How, and why? Because we rob it of its red corpuscles, which are diminished in quantity by such means more rapidly than the other constituents. I hold that this is particularly deleterious and expensive to the constitution, because they constitute that portion of the blood that possesses the calorific and vivifying powers. They contain the globulin, hematin, and the principal portion of the phosphorus, fat, and potash salts, the material especially prepared by the blood-cells, and used so largely in the construction and nutrition of the nervous and muscular fabrics; robs the vital fluid of its nutrient and plastic materials; and, when we remember that the functions of the blood are, first, to maintain the activity of the nervous and muscular system, and, secondly, to supply the the materials for the molecular changes constantly going on in the tissues, we can readily understand why its withdrawal so frequently produces that peculiar condition of the brain and nervous system marked by great weakness and extreme irritability. Arterial blood is a powerful stimulant to the brain, while venous blood is equally as powerful a sedative. Hence, we can account for the convulsions in children sick with pneumonia, upon the theory that they are suffering from an overdose of a powerful sedative; so, also, upon the same theory, we must account for the extreme prostration that often ensues in adults sick with pneumonia. There is a lack of stimulation of the brain by oxygen. The blood in this disease is already deficient in quality, by reason of imperfect aëration—is at least five per cent. deficient in oxygen, in consequence of obstructed respiration and pulmonary circulation. Then, to take from it its globulin, hematin, and other nutrient materials by general blood-letting, certainly seems to me to be an illogical and an irrational procedure. Again, we all know that even if we do gain any advantage over the congestion by reducing the quantity of blood in circulation, it is but for a short time, for the reason that the effects of blood-letting are not as lasting upon the quantity of blood in circulation as upon the quality. The quantity is soon made up by absorption from the watery portions of the tissues, so that the volume does not long remain diminished, and the heart soon has as large a quantity to propel, and the already weakened lung an inferior article to aërate, because it has been diluted by bleeding, and is, therefore, poorer, less nourishing, less stimulating, and less capable of sustaining the vital powers of life. This being the fact, *I say blood-letting in pneumonia is too expensive to the constitution, and the advantages gained over the disease are not equal to the waste.*

I am aware that in pneumonia the tendency towards degeneration is promoted by the disordered circulation in the lung. But is it claimed by its advocates that bleeding from the vein in the arm has any direct effect upon the coagulated extravasations and exudations of inflammation in the lung? I apprehend not. On the contrary, as I understand, the objects of the abstraction of blood by general blood-letting, are to ease the gorged capillaries of the inflamed organ by first reducing the quantity of blood in circulation, and, secondly, by lessening the heart's action. My proposition is, that the treatment should comprise the attempt to remove or counteract such local derangement, by the employment of such means as will not compromise the constitutional powers of the patient. Now, we know that bleeding does not always arrest the inflammatory action. On the contrary, there is great danger that it may continue, and even be increased and extended, in spite of the bleeding. In such cases, at least, there is great danger that the strength of the system will be so reduced as to prove unequal to the process of restoration; for, to remove the interstitial extravasation and to repair the damage that has occurred, a certain degree of vital power is requisite, together with a sufficient quantity of healthy blood.

Even when the bleeding does arrest the inflammatory action, it will not always cure the effects of the inflammation; it may be, and, in my opinion, is, liable to render them lingering in their departure, or even increase, and determine their fatality.

The subsequent debilitating effects of the loss of blood upon the system may be more certain and hurtful than the effects of the bleeding upon the local inflammation is beneficial. In selecting our remedies in the treatment of pneumonia, we should always bear in mind the fact that acute pneumonia, marked by no anomaly, complicated with no other malady, and occurring under ordinarily favorable circumstances, generally tends towards recovery, resolution, and a favorable issue. I say *generally*, but it does not always result thus favorably. In some cases, it assumes, from the very commencement, an adynamic or an otherwise unfavorable character, unwonted and accidental symptoms manifest themselves in its progress, owing to various contingent causes operating during its continuance, causes proper to the individual affected, or to a peculiarity of the nature and combination of the external agents which produce it. Age, sex, constitution, locality, epidemic influences, state of the season, atmosphere, or the epidemic constitution upon which it supervenes all tend to complicate and change the natural tendency of the disease. They should all be looked after and taken into account, before we apply or decide upon our remedy. *We must treat every case according to its own individuality.*

In no disease, perhaps, is this more clearly and impartially demanded, than in pneumonia. I say, then, it is especially important that we keep in mind, while prescribing in this disease, the fact that, in a majority of cases, the disease will get well in its own natural course, when not complicated. And, again, that it has two distinct periods, with distinct symptoms and indications of treatment—the period of congestion, high febrile action, and that of hepatization and disorganization.

If we had no recorded evidence, no statistics, no experience, nothing except general principles to fall back upon, even then, in my judgment, blood-letting could only be considered a logical remedy in the very commencement of a few exceptional cases, only admissible in certain sthenic cases and robust persons. But, most happily, we are not thus devoid of recorded evidence—statistical proofs against the use of the lancet in this disease. The French physicians, who bleed often and copiously enough to satisfy the most bloodthirsty, lost a very large per cent. of their patients. Experience has proved, most undeniably, that even the entire expectant plan of treatment has saved a larger per cent. of patients than the most copious bleeders. Do not understand me, however, as arguing for, or adopting, the entire expectant plan of treatment—that is too extreme.

I would apply such measures as will *best assist nature*, without compromising the vital properties of the blood, or the strength of the patient. I would select my remedy with direct reference to indications of the case, and not because it is pneumonia. If I found quick, hurried, and labored breathing, full pulse, and other indications of congestion of the lung, I should at once attempt to counteract the difficulty. I should apply hot fomentations and counter-irritants to the chest and extremities, or even to the whole external surface, if necessary; I would invite the blood to the surface; I would, at the same time, administer such arterial sedatives as *are known to control the heart's action promptly*, without robbing the vital current of its nutrient elements. My experience is, that by reasonable energy and perseverence we can accomplish these desirable objects without much delay. Enveloping the chest with cotton batting, covered with oil-silk, has a fine influence upon the capillary circulation about the chest. When we have once controlled the heart's action, we can place a sentinel over the pulse *who can say with full confidence of being obeyed, so fast and no faster*, and if he perform his duties well and faithfully, we may confidently expect the patient to have a speedy convalescence, *because he has only to recover from the disease*, and not from what is often *harder*, *the bleeding*.

Perhaps, you will naturally inquire, with what particular article of the materia medica I propose to control the heart's action? I shall answer, with veratrum viride. I regard it as the most valuable arterial and nervous sedative at our command. I prefer it to the tartar emetic in pneumonia, because it is less violent in its action, and less exhausting to the constitution. It seems to be particularly adapted to the treatment of inflammation of the lungs. It controls the heart's action promptly, and thus relieves the congestion, and, if resorted to early, will certainly obviate the use of the lancet. Nor need we be surprised at this, when we consider that veratrum viride affects the system, primarily, through the pneumogastric, and, secondarily, through the sympathetic nerves. Thus, we can easily and rationally account for its prompt action upon the heart, and general arterial system, and, through them, upon parts supplied by the solar plexus, while the brain remains but slightly, if at all, affected. In small doses, at long intervals, whereby the system is constantly stimulated to reaction, it is not cumulative, but exercises an undoubted eliminating and depurating influence over the blood and the glandular system, removing obstructions, abating congestions, relieving nervous irritation dependent thereon, and promoting the venous circulation. When we so gradually increase the dose as to control the action of the heart, but not to destroy the balance between the venous and arterial circulation, then it becomes a most valuable therapeutic in acute inflammations. This gradual controlling the heart's action is very important in pneumonia. When we recollect the valuable and interesting experiments of T. WHARTON JONES upon the tail of a tadpole, under the microscope, where he found that when the *vis a tergo* of an artery is suddenly diminished, congestion of the capillaries and venous radicals to which the artery leads is established, then we can appreciate the force of the proposition. Here is where I claim another advantage in favor of veratrum viride over blood-letting. In bringing the system so suddenly under the influence of the loss of blood, as is recommended, we arrest the *vis a tergo* of the arterial system, and the balance between the venous and arterial circulation is destroyed, and congestion of the capillaries and venous radicals of the lungs liable to ensue. The veratrum viride, at the same time that it controls the heart's action, also acts as a powerful diaphoretic, diuretic, and expectorant. Under its use we have profuse colliquative sweating, an increased secretion of bile, urine, and a copious exudation along the respiratory passages, all of which are especially calculated to unload the capillaries and venous radicals of their surplus blood. But, in using this remedy, we should remember, while applying it in acute inflammations, our object is to prevent, rather than to promote, exudations; therefore, bringing the system under its influence gradually is the only way warranted by its physiological effects.

Finally, in conclusion, I desire to urge, that since blood-letting robs the blood of its vivifying and nutrient materials, by removing its red corpuscles, its globulin, hematin, phosphorus, fat, and potash salts, it compromises the constitutional powers of the patient; and, inasmuch as we possess other equally efficacious remedies, wherewith we can accomplish the same results equally quick, without thus robbing the vital current of its life-supporting properties, therefore, I say blood-letting, in uncomplicated cases of pneumonia, *is unnecessary*, *uncalled for*, *and can only be justifiable in very rare and extreme cases.*

